# Comparative Transcriptome Reveals the Genes’ Adaption to Herkogamy of *Lumnitzera littorea* (Jack) Voigt

**DOI:** 10.3389/fgene.2020.584817

**Published:** 2020-12-08

**Authors:** Ying Zhang, Yukai Chen, Yan Zhou, Jingwen Zhang, He Bai, Chunfang Zheng

**Affiliations:** ^1^School of Life Sciences and Technology, Lingnan Normal University, Zhanjiang, China; ^2^National and Local Joint Engineering Research Center of Ecological Treatment Technology for Urban Water Pollution, College of Life and Environmental Science, Wenzhou University, Wenzhou, China; ^3^Ministry of Education Key Laboratory for Ecology of Tropical Islands, College of Life Sciences, Hainan Normal University, Haikou, China

**Keywords:** transcriptome, *Lumnitzera littorea*, floral organ, MADS-box, mangrove

## Abstract

*Lumnitzera littorea* (Jack) Voigt is among the most endangered mangrove species in China. The morphology and evolution of *L. littorea* flowers have received substantial attention for their crucial reproductive functions. However, little is known about the genomic regulation of flower development in *L. littorea*. In this study, we characterized the morphology of two kinds of *L. littorea* flowers and performed comparative analyses of transcriptome profiles of the two different flowers. Morphological observation showed that some flowers have a column embedded in the petals while others produce a stretched flower style during petal unfolding in flowering. By using RNA-seq, we obtained 138,857 transcripts that were assembled into 82,833 unigenes with a mean length of 1055.48 bp. 82,834 and 34,997 unigenes were assigned to 52 gene ontology (GO) functional groups and 364 Kyoto Encyclopedia of Genes and Genomes (KEGG) pathways, respectively. A total of 4,267 differentially expressed genes (DEGs), including 1,794 transcription factors (TFs), were identified between two types of flowers. These TFs are mainly involved in bHLH, B3, bZIP, MYB-related, and NAC family members. We further validated that 12 MADS-box genes, including 4 MIKC-type and 8 M-type TFs, were associated with the pollinate of *L. littorea* by herkogamy. Our current results provide valuable information for genetic analysis of *L. littorea* flowering and may be useful for illuminating its adaptive evolutionary mechanisms.

## Introduction

*Lumnitzera littorea* (Jack) Voigt. (Combretaceae, *Lumnitzera* genus) is a non-viviparous Indo-West Pacific mangrove species. *L. littorea* is sparsely distributed in India, Sri Lanka, Myanmar, Thailand, Malaysia and Indonesia, and China (Zhou et al., 2018). Based on IUCN (International Union for Conservation of Nature) Red List Categories and Criteria, *L. littorea* was listed as a least concern (LC) species (Polidoro et al., 2010). In China, the wild plant number was only 359 in 2006, and rapidly declined to 9 in 2018. The narrow distribution of it was only in Sanya Tielu harbor and Lingshui Dadun village of Hainan Island. In 2018, all of the wild *L. littorea* growing in Lingshui Dadun village died (Fan and Chen, 2006; Zhang et al., 2018). During 13 years of field observation, no seedlings or young trees were observed due to high (76%) seed abortive rate (Zhang et al., 2017). The protection of this species is facing a great challenge and the mangrove *L. littorea* is therefore is listed as a plant under state protection (category II) (Zhong et al., 2011; Zhang et al., 2013).

*L. littorea* has relatively low genetic diversity and gene flow in China (Su, 2004), possibly because of the limited number of wild individuals and distribution area (Zhang et al., 2018). According to the pollen-ovule ratio (P/O) and hybridization index (OCI) analyses, *L. littorea* is classified as a typical cross-pollinated plant with red petals and erectly terminal inflorescence. Most of the *L. littorea* flowers can only be pollinated from the same tree or even from the same flower (Li et al., 2016; Zhang et al., 2016, 2017). The pollen viability *L. littorea* in China was lower than 10% (Zhang et al., 2013, 2016). The heavy abortion of *L. littorea* seeds resulted from the high empty embryo rate (Zhang et al., 2018). In woody perennials, there are several studies on the regulation of flowering (Chen et al., 2018), but the underlying molecular mechanisms of floral dynamics and breeding systems in the development of *L. littorea* remain poorly understood.

MADS-box transcription factors play crucial roles in floral organ formation, embryo and reproductive development and flowering time control (Angenent, 1995; Moon et al., 2003; Arora et al., 2007; Irish, 2010; Mohanty and Joshi, 2018; Zhang et al., 2018). Extensive studies of *Arabidopsis* mutants show several genetic models of floral organ formation (Coen and Meyerowitz, 1991; Theissen et al., 2016). The ABCDE model involves five subgroups of the MADS family. *AP1* (*APETALA 1*) and *AP2* belong to A-class; *AP3* and *PI* (*PISITTALA*) belong to B-class; *AGAMOUS* (*AG*) belongs to C-class; *SEEDSTICK* (*STK*) belongs to D-class; and *SEP1* (*SEPALLATA 1*), *SEP2, SEP3*, and *SEP4* belong to E-class (Bowman and Meyerowitz, 1991). The combinations of MADS-box proteins determine the tetrameric complexes (Theiβen and Saedler, 2001). For instance, class A+B+E genes control petal development, B+E+C genes determine stamen development, C+E specify carpels, and D+E are necessary for ovule development (Wellmer et al., 2014). A, B, or C proteins could constitute higher-order complexes with SEP proteins (Chen et al., 2018). The *sep1/2/3/4* mutant displays indeterminate flowers composed of leaf-like organs and sepal development, indicating the role of SEP proteins in control flower development (Favaro et al., 2003). Importantly, the “ABCDE” model key genes are conservative in the control of petal and style development in *Soybean*, *Impatiens* and *Marcgravia* (Geuten et al., 2006; Litt and Kramer, 2010; Huang et al., 2014).

TF flowering locus C is a convergence point for environmental and endogenous pathways that regulate flowering time in *Arabidopsis* (Mateos et al., 2015). *AGL27* mutants flower earlier in a dosage dependent manner while transgenic plants carrying *AGL27* overexpression cassettes are delayed in flowering (Scortecci et al., 2001; Yun et al., 2011). FLC interacts with another MADS-box protein, SHORT VEGETATIVE PHASE (SVP), to delay flowering (Li et al., 2008). The function of the MADS-box gene has been verified in the flower development of many species, but it has not been reported in *L. littorea*.

Here, we conducted *de novo* transcriptome sequencing of two kinds of flowering behavior with different types of style development for *L. littorea* in order to investigate gene expression patterns associated with special style development morphology. To our knowledge, this is the first comprehensive transcriptomic study of flower development for *L. littorea*, providing important bioinformatic resources for the investigation of genes involved in flower development, and building a foundation for investigating the role of these genes and gene networks in the evolution of floral diversity across *L. littorea.*

## Materials and Methods

### Plant Material

The *L. littorea* trees live in Sanya Tielu Bay, Hainan, China (18°15′-18°17′N, 109°42′-109°44′E). Flowers with columns embedded in the petals (L-1) or with stretched styles (L-2) were collected from one florescence of one tree at 9 am on July 20, 2017. For each type, at least three flowers were selected. The materials were immersed into liquid nitrogen and stored at −80°C for subsequent research. Three biological replicates were prepared for sequencing.

### RNA Extraction and Deep Sequencing

RNA isolation was performed with TRIzol^®^ Reagent (Invitrogen, United States) following the manufacturer’s instructions. RNA samples with an RNA integrity number (RIN) >9.5 were used for purification and subsequent cDNA construction with the TruSeq RNA sample RNA prep kit (Illumina, United States). After synthesis of the first-strand cDNA, the second-strand cDNA was produced using buffer, dNTPs, RNase H, and DNA polymerase I. The double-strand cDNA was purified using the QIAquick PCR extraction kit (QIAGEN, Germany) and washed with EB buffer for end repair and single nucleotide adenine (A) addition. After PCR amplification for 15 cycles, the products were loaded onto flow cell channels at 12 pM for paired-end 150 bp × 2 sequencing with the Illumina HiSeq 4000 platform (Majorbio, Shanghai, China).

### *De novo* Assembly and Analysis of Illumina Reads

Clean reads were obtained by (1) removing the adapters and reads without fragmentation; (2) cutting the low quality bases (quality score less than 20) at the 3′ end of the sequence and then, if the quality of the residual sequence is still less than 10, removing the entire sequence, while sequences with a quality greater than 10 are retained; (3) removing reads that contain too many Ns (≥10%); and (4) removing reads less than 20 bp long after adapter discarding and quality control. Analysis tools: SeqPrep^1^ and Sickle^2^. The *de novo* assembly was conducted with the Trinity software^3^ (Grabherr et al., 2011). The raw data have been uploaded to NCBI SRA under accession numbers SRR6429108 to SRR6429113.

### Transcriptome Annotation

BlastX was used to perform sequence alignments between the transcriptome and sequence data from the NR, String, SwissProt and Kyoto Encyclopedia of Genes and Genomes (KEGG) databases. Alignments with *E*-values less than 1e^–5^ were chosen. NCBI_NR is a collection of sequences from several sources, including translations from annotated coding regions in GenBank, RefSeq and TPA, as well as records from SwissProt, the Protein Information Resource (PIR), the Protein Research Foundation (PRF), and the Protein Data Bank (PDB). Via GO (gene ontology) annotation, the database standardizes the biological terms of genes and gene products and unifies the definitions and descriptions of gene and protein functions (Grabherr et al., 2011). The Clusters of Orthologous Groups of proteins (COG) database^4^ is an orthologous protein cluster database that depends on the phylogenetic relationships of complete protein sequences from 66 selected strains. Functional annotation, classification and protein evolution analysis can be performed by comparing sequences with the COG database (Zhang et al., 2014). Pathway assignments were performed according to the KEGG^5^ pathway database (Grabherr et al., 2011; Huang et al., 2015) with BlastX and an *E*-value threshold of 1e^–5^.

### Identification of Differentially Expressed Transcripts

EdgeR^6^ was used for differential expression analysis. Gene read count data were calculated as the input of EdgeR or DESeq2. This analysis method is based on the negative binomial distribution model. The screening criteria of significant DEGs were as follows: FDR < 0.05 and |log2FC| > = 1 (Anders and Huber, 2010).

### Annotation and Phylogenetic Analysis

To identify the TFs represented in the *L. littorea* transcriptomes, all DEGs were searched against the plant TF database PlantTFDB 4.0 (Jin et al., 2014)^7^. BlastN searches of the Phytozome database, using *Arabidopsis* genes as queries, were used to identify flower and floral organ development-related genes in *L. littorea*. The CDS sequences of all MADS-box genes from *Arabidopsis*, *Hevea brasiliensis*, and *Fragaria ananassa* were downloaded from GenBank. Multiple sequence alignments and the phylogenetic analysis of CDS sequences were performed as described previously (Cheng et al., 2017). An unrooted phylogenetic tree was created with the neighbor-joining method by using MEGA-X, and a bootstrap test was set to 1000 replicates (Kumar et al., 2018).

### Real-Time PCR Analysis

Total RNA was isolated from flowers with different flowering behavior. Three biological replicates were set. qRT-PCR assays were conducted using the ABI PRISM 7300 Sequence Detection System (Applied Biosystems) with SYBR Green PCR Master Mix (Applied Biosystems). The housekeeping gene *ACTIN* (c14139_g1) was used for normalization in each qRT-PCR run. The relative expression levels of target genes are presented as 2^–ΔΔ*CT*^ (Livak and Schmittgen, 2001). Primers used in this study are listed in Supplementary Table S1.

## Results

### Floral Structure Morphogenesis of *L. littorea* Flowers

**FIGURE 1:**
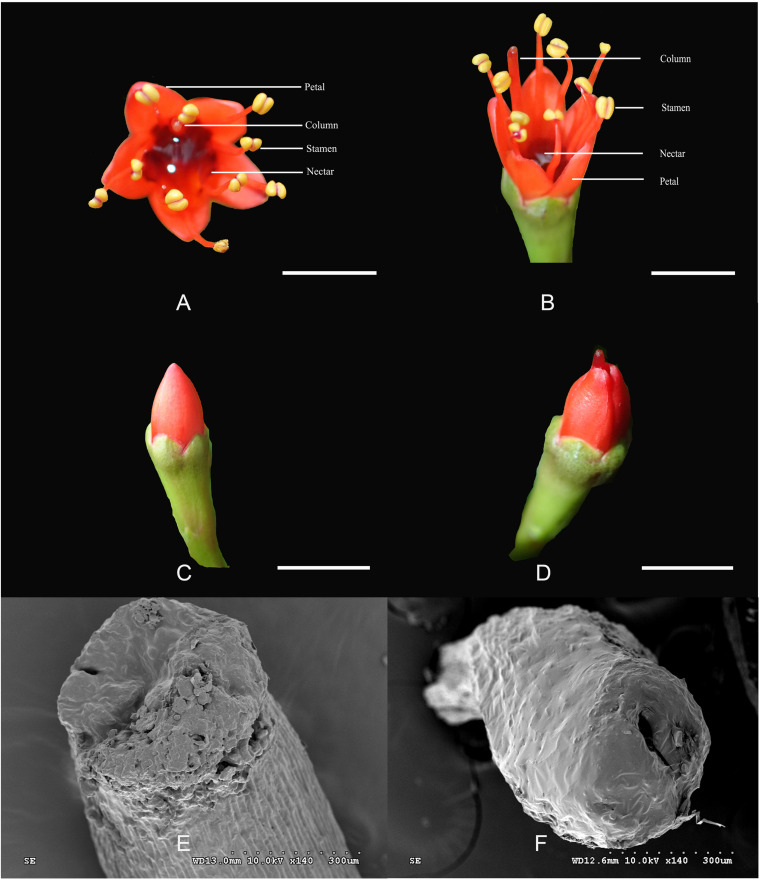
The structure of flowers in *Lumnitzera littorea*. **(A,B)** Hermaphroditic flower; **(C)** L-1: column into a petal; **(D)** L-2: column stretched beyond the petals; **(E)** No pistillar chord on stigma; **(F)** Pistillar chord on stigma.

The *L. littorea* flowers are hermaphroditic with red, erect petals and a deep, curved calyx tube with abundant nectar (Figures 1A,B). The diameter of a single flower is approximately 6.70 ± 0.04 mm. The five petals per flower are 4.20 ± 0.43 mm long. The pistils and stamens are approximately the same length and as long as 8 mm. Stamens are prominently exserted at anthesis after the stamens unfold. During flower opening, two kinds of flowers can be found before the petals are uncovered. One kind retains stigma within the petals and was named as L-1 (Figure 1C); the other kind (L-2) are herkogamy flowers and has columns stretched beyond the petals before flowering (Figure 1D). In L-1, no stylar canal was found on the stigma (Figure 1E). In L-2, there is an obvious stylar canal in the stigma (Figure 1F), the filaments are kept folded, and the anthers are kept intact.

### RNA-Seq and *de novo* Assembly

To obtain an overview of the transcriptome profiles, L-1 and L-2 were sampled at different column development stages for Illumina deep sequencing. 66.83 (L-1) and 65.40 (L-2) million raw reads were yielded. A total of 138,857 transcripts with a GC percent of 38.91%, average length of 1665.35 and an N50 size of 3049, were obtained (Table 1). 82,833 unigenes with a GC percent of 38.59, an average length of 1055.48 and an N50 size of 2270, were produced.

**TABLE 1:** Summary of Illumina transcriptome sequencing.

Type	Unigene	Transcripts
Total sequence number	82833	138857
Total sequence base	87428846	231245876
Percent GC	38.59%	38.91%
Largest	17599	17599
Smallest	201	201
Average unigene length (bp)	1055.48	1665.35
N50 length (bp)	2270	3049

**TABLE 2:** MADS-box gene names and attributes of *Lumnitzera littorea*.

Gene Name	Transcriptome ID	Strand	Protein length	Subfamily
LliMADS1	c14522_g1_i1	−	175	AP3
LliMADS2	c16514_g1_i1	−	175	SVP/AGL24
LliMADS3	c17232_g1_i1	+	175	Mα
LliMADS4	c17574_g1_i1	−	175	SVP/AGL24
LliMADS5	c19549_g1_i1	+	165	MIKC*
LliMADS6	c19801_g1_i3	−	122	MIKC^*c*^
LliMADS7	c21067_g7_i3	−	104	SVP/AGL24
LliMADS8	c23460_g1_i1	−	173	MIKC*
LliMADS9	c24786_g2_i1	−	174	AP3
LliMADS10	c25298_g6_i2	+	174	SEP
LliMADS11	c44184_g1_i1	−	157	Mα
LliMADS12	c44474_g1_i1	+	142	Mγ

### Functional Annotation of Unigenes

The unigene sets obtained from the *L. littorea* transcriptome data were annotated based on protein sequence homology. All transcripts and unigenes produced were searched against the NCBI NR, SwissProt, String, KEGG and Pfam databases with an *E*-value threshold < 1e^–5^. As a result, 138,857 transcripts and 82,833 unigenes were annotated (Supplementary Figure S1). The similarity distribution analysis identified 41,687 transcripts and 13,958 unigenes that exhibited high sequence similarity (from 80% to 100%) with known gene sequences. Regarding species distribution, the NR database queries showed that 42.6% of the *L. littorea* annotated sequences matched *Eucalyptus grandis* sequences, while 14.14, 13.52, and 8.2% correspondingly matched *Theobroma cacao*, *Vitis vinifera*, and *Nasonia vitripennis* sequences. Characteristics of the homology search of *L. littorea* unigenes against the NR database are shown in Supplementary Figure S2.

Based on the BLASTX results against the NR database, we assigned GO terms to the assembled unigenes to obtain GO functional annotations and categorizations. All of the unigenes were used to query the GO database in order to classify their predicted functions (Supplementary Figure S3 and Supplementary Table S2). In the “biological process” category (34,918 unigenes), macromolecule metabolic process (4,389) was the largest subcategory. In the “cell component” (31,040 unigenes) and “molecular function” (16,876 unigenes) categories, intracellular (4,429) and nucleotide binding (2,755) were the most abundant GO terms, respectively. The GO analysis indicated that a high number of unigenes were associated with the various biological processes and molecular functions in *L. littorea* floral tissues.

The annotated sequences were further applied to a search against the clusters of orthologous groups of proteins (COG) and clusters of orthologous groups for eukaryotic complete genomes (KOG) databases for functional prediction and classification. As a result, each annotated unigenes was assigned 25 COG and 25 KOG terms. Among the assigned terms, the three most highly represented categories in the two databases were identical. (1) general function prediction only (883 unigenes in the COG databases; 1267 unigenes in the KOG databases); (2) signal transduction mechanism (844 unigenes in the COG databases; 1136 unigenes in the KOG databases); and (3) posttranslational modification, protein turnover, and chaperones (740 unigenes in the COG databases; 1006 unigenes in the KOG databases). The smallest group was “cell motility,” with 3 unigenes in the COG databases and 1 unigene in the KOG databases (Supplementary Figure S4).

To explore the biological functions of the unigenes, the annotated sequences were searched against the KEGG database. 42.3% (34,997/82,833) of unigenes were assigned to 364 KEGG pathways. The top five pathways were “carbon metabolism” (ko01200), “ribosome” (ko03010), “protein processing in endoplasmic reticulum” (ko04141), “biosynthesis of amino acids” (ko01230) and “oxidative phosphorylation” (ko00190) (Supplementary Figure S5). These results provide valuable information for gene discovery and functional characterization.

### Comparation of DEGs Between Two Types of *L. littorea* Flower

To examine gene expression among two different floral behaviors, two transcriptome profiles were compared. We found 4,267 distinct unigene sequences that were significantly different between L-1 and L-2. Of these, 1,874 were upregulated and 2,393 were downregulated in herkogamy flowers (L-2) (Supplementary Table S3). Floral homeotic protein DEFICIENS-like gene (c44184_g1), SVP-like floral repressor gene (c16514_g1), receptor-like kinase in flowers (c18462_g3), MYB family genes (MYB16, c24005_g10; MYB86-like, c22851_g2; MYBP, c14148_g1, c22970_g1, c16951_g1; MYB1R1, c20799_g1; R2R3-MYB, c14888_g1; MYB32-like, c14253_g1; MYBJ6, c2873_g1; MYB124, c22682_g1; MYB-like protein, c23232_g17, and MYB5, c10786_g1), MADS-box family genes (STAMADS11, c21067_g7; K-box, c14522_g1, and MADS-box 24?, c25298_g6) and embryo development related genes (MEDEA 18-1, c24129_g1 and LEA, c23402_g1). The GO annotation analysis classified groups of genes with significantly differential expression into three categories: the biological process, cellular component and molecular function categories (Supplementary Figure S6). To identify the unigenes involved in metabolic or signal transduction pathways that were significantly enriched, all of the DEGs were used to query the KEGG database. A total of 1,215 pathways from the KEGG database were enriched (Supplementary Table S4). These significant pathways were classified into environmental information processing (EIP), genetic information processing (GIP), cellular processes (CP) and metabolism (M) categories. The top three significant pathways were “flavonoid biosynthesis” (KO00941), “plant hormone signal transduction” (KO04075) and “sesquiterpenoid and triterpenoid biosynthesis” (KO00909) (Supplementary Figure S7).

### Transcription Factors in DEGs Modulating the Herkogamy of *L. littorea* Flowers

**FIGURE 2:**
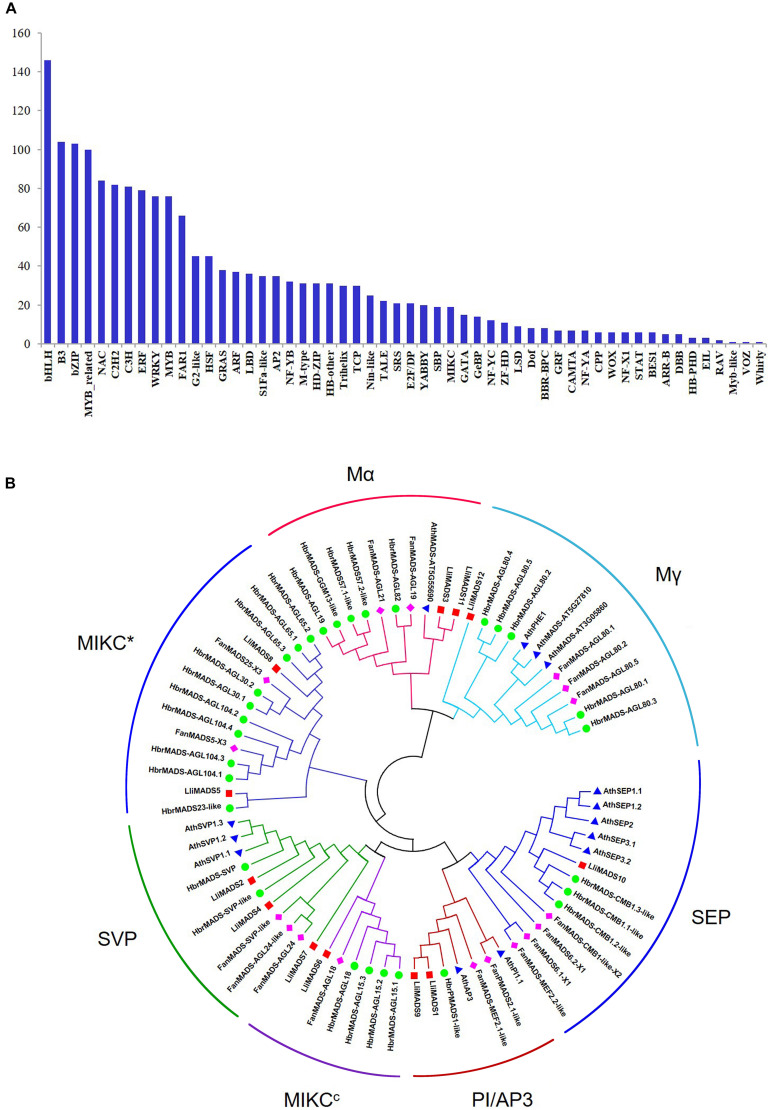
Identification of TFs and MADS-box genes in DEGs of *Lumnitzera littorea.* **(A)** Classification of TF families. **(B)** Phylogenetic relationships of MADS-box TF CDSs from *L. littorea*, *Arabidopsis*, *Fragaria ananassa*, and *Hevea brasiliensis*. The unrooted phylogenetic tree was created with MEGA-X by the neighbor-joining method, and the bootstrap test was performed with 1,000 iterations. Red, blue, green, and purple dots indicate *L. littorea*, *Arabidopsis*, *Hevea brasiliensis*, and *Fragaria ananassa* genes, respectively. The outer circle shows the identified subfamily in MADS proteins.

Transcription factors (TFs) play key regulatory roles in floral development by binding to specific motifs in the promoters of target genes (Chen et al., 2018). Here, we showed that 41% (1,749/4,267) of DEGs between L-1 and L-2 belong to TFs (Supplementary Table S4). These TFs were classed into 54 categories with bHLH, B3, bZIP, MYB-related, NAC, C2H2, C3H, ERF, WRKY, and MYB being the most highly represented (Figure 2A). It is noted that the *MADS*, *bZIP*, *bHLH*, and *MYB* genes play key roles in the regulation of flower development and flowering time (Streisfeld et al., 2013; Wang et al., 2013; Rocheta et al., 2014).

### Phylogenetic Analysis of MADS-Box Genes Associated With the Herkogamy of *L. littorea* Flowers

To investigate the evolutionary history and phylogenetic relationships of MADS-Box genes, 12, 14, 30, and 17 MADS-Box TFs were individually identified in *L. littorea, Arabidopsis*, rubber tree, and strawberry based on the PlantTFDB 4.0 database (Figure 2B and Supplementary Table S5). A neighbor-joining phylogenetic tree was then generated by alignment of these MADS-box proteins. As shown in Figure 2B, these proteins were classified into seven groups, similar to the description in a previous report (Parenicova et al., 2003). In each subgroup, MADS proteins in *L. littorea* were more closely related to those in rubber tree, except in the Mα subfamily. Twelve differentially expressed MADS proteins between L-1 and L-2 appeared in each putative functional group. Of them, *LliMADS3* and *LliMADS11* are the most homologous with AT5G55690, and belong to the Mα subdivision of type I MADS-box genes. *LliMADS12* and *AthPHERES1* (*PHE1*) are on the same branch in the Mγ subdivision of type I MADS-box genes. *LliMADS1* and *LliMADS9* belong to the B class of the AP3 subfamily, and *LliMADS10* belongs to the SEP subfamily.

### RNA-Seq Expression Validation by Real-Time PCR

**FIGURE 3:**
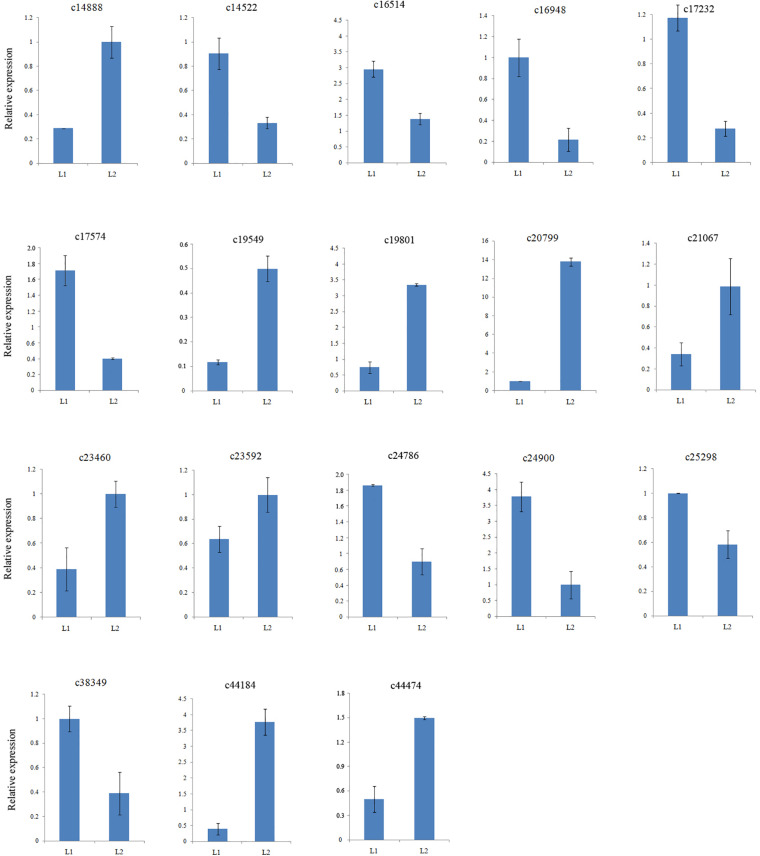
Validation of assembled unigenes by qPCR.

To confirm the gene expression patterns identified by RNA-Seq data, the transcript levels of twelve MADS-box genes together with six other DEGs were examined by qRT-PCR. All 18 selected DEGs were successfully amplified with single bands of the expected sizes. The expression of 10 genes were down regulated and that of 7 genes were up regulated (Supplementary Table S6), consistent with those of the RNA-Seq data (Figure 3). Therefore, the DEGs obtained from the assembled transcriptome were accurate and reliable.

## Discussion

*Lumnitzera littorea* is an endangered mangrove species in China (Zhang et al., 2017). Herkogamy is found in almost 60% of *L. littorea* flowers, but approximately 40% of the flowers have a column embedded in the petals when the petals unfold during florescence. Almost all those flowers have empty seeds, which are speculated by the results of forced self-pollination. Thus, the breeding system of *L. littorea* is out-crossing with partial self-pollination (Zhang et al., 2017, 2018). Out-crossing is obligate in unisexual flowers and selfing can occur in hermaphrodite flowers, but a self-compatible level can be strongly selected by herkogamy, i.e., the spatial separation of anthers and stigmas within a flower (Mertens et al., 2018). In addition, geitonogamous selfing is not prevented within or between inflorescences on a plant when flowers are at different sexual phases in *L. littorea* (Zhang and Wolfe, 2016; Zhang et al., 2017). The temporal separation of male and female phases is a common floral feature in hermaphrodite species (Rosas-Guerrero et al., 2017). In such a bad survival trend, only the herkogamy flower morphogenesis of *L. littorea* could hardly improve the natural reproduction rate. Therefore, flower morphogenesis suitable for geitonogamous selfing as L-1 may be a positive adaption for this survival condition, even though most of them failed in seed production.

In the present study, we analyzed the transcriptome profiles of flowers with columns embedded in the petals (L-1) and with stretched styles (L-2) and identified a total of 82,833 unigenes and 138,857 transcripts, respectively. Using the stringent criteria of both FDR < 0.05 and |log2FC| > = 1, we detected 4,267 unigenes that were significantly different between L-1 and L-2. These results imply a diverse and complex mechanism of column development gene expression in *L. littorea*.

Gene ontology functional enrichment revealed that a high number of genes were associated with various biological processes and molecular functions in *L. littorea* floral tissues. KEGG pathway analysis showed that many DEGs were involved in secondary metabolite biosynthesis, including carbon metabolism, ribosome, protein processing in the endoplasmic reticulum and amino acid biosynthesis. Furthermore, plant oxidative phosphorylation that plays an important role in plant floral development (Liu et al., 2019) is also activated.

TFs play important roles in the regulation of flower development and flowering time (Li et al., 2017). In this study, we identified 1,749 differentially expressed TFs with 54 diverse categories. The different expression of these TFs indicated their possible different roles in modulating the formation of herkogamy flowers in *L. littorea*. Of these TFs, MADS-box family genes function in flower development (Parenicova et al., 2003). MADS-box proteins are generally divided into types I and II. Type I is categorized into Mα, Mβ, Mγ, and Mδ clades and type II is classified into MIKC^*c*^ and MIKC^∗^ (Mohanty and Joshi, 2018). Several type I MADS box TFs are shown to be involved in reproductive development in *Arabidopsis* (Luo et al., 2008). The empty embryo rate of 70% may be related to the upregulated expression of Mγ genes in the flowers of *L. littorea* in China. *SVP* has been identified to delay flowering by modulating the biosynthesis of gibberellin in *Arabidopsis* (Andrés et al., 2014) and *Jatropha curcas* (Hui et al., 2018). In the flowers of *L. littorea*, two downregulated *SVP* genes (*LliMADS2* and *LliMADS4*), and one upregulated *SVP* genes (*LliMADS7*) affect dichogamy morphogenesis, which may also be caused by the modulating the biosynthesis of gibberellin in florescence. In *Arabidopsis*, the petal, stamen and carpel of *sep1sep2sep3* mutants are switched to sepal (Ditta et al., 2004). *LliMADS10* and *AthSEP* were found in the same branch covering SEPs (Figure 2B). In the flowers of *L. littorea*, *LliMADS10* was downregulated and may contribute to dichogamy morphogenesis. These genes should be paid more attention in further research.

## Data Availability Statement

The datasets presented in this study can be found in online repositories. The names of the repository/repositories and accession number(s) can be found below: https://www.ncbi.nlm.nih.gov/, SRP127706.

## Author Contributions

YZa and CZ designed the study and modified the manuscript. YZa, YC, YZo, JZ, and HB collected the samples and acquired the data. YZa and YC drafted the manuscript. CZ, JZ, and HB helped to draft the manuscript. All authors read and approved the final manuscript.

## Conflict of Interest

The authors declare that the research was conducted in the absence of any commercial or financial relationships that could be construed as a potential conflict of interest.

## Footnotes

1 https://github.com/jstjohn/SeqPrep

2 https://github.com/najoshi/sickle

3 https://github.com/trinityrnaseq/trinityrnaseq/releases

4 http://www.ncbi.nlm.nih.gov/COG/

5 http://www.genome.jp/kegg/

6 http://www.bioconductor.org/packages/2.12/bioc/html/edgeR.html

7 http://plantregmap.gao-lab.org/

## Supplementary Material

Supplementary Figure 1 Transcript and unigene annotation with the NCBI NR, SwissProt, String, KEGG and Pfam databases of *L. littorea* transcriptome data.

Supplementary Figure 2 Species distribution with BLAST hits to the annotated unigenes of *L. littorea*.

Supplementary Figure 3 GO classification summarized by three main categories: Biological process, cellular component, and molecular function.

Supplementary Figure 4 COG and KOG functional classification of the unigenes of *L. littorea* with NR annotation.

Supplementary Figure 5 The biological pathways of *L. littorea* flower unigenes against the KEGG database.

Supplementary Figure 6 DEGs in different column development stages between L-1 and L-2.

Supplementary Figure 7 Functional KEGG pathway annotation of the DEGs of *L. littorea*.

Supplementary Table 1 Primers used in this study.

Supplementary Table 2 Classify the predicted functions of the query GO database.

Supplementary Table 3 DEGs in the materials L-2 vs. L-1.

Supplementary Table 4 KEGG pathway enrichment analysis.

Supplementary Table 5 Sequence of the MADS-Box genes in *L. littorea*.

Supplementary Table 6 Validation of assembled unigenes by RNA-Seq.

## Abbreviations

bZIP: basic leucine zipper
COG: clusters of orthologous groups of proteins
DEGs: differentially expressed genes
ERF: ethylene-responsive factor
FLC: TF flowering locus C
GO: gene ontology
HLH: helix-loop-helix
NR: RefSeq non-redundant proteins
KEGG: Kyoto Encyclopedia of Genes and Genomes
MYB: v-myb avian myeloblastosis viral oncogene homolog
PDB: protein data bank
MADS: mini chromosome maintenance 1, agamous, deficiens, and serum response factor
PIR: protein information resource
PRF: protein research foundation
SEP: sepallata
TFs: transcription factors.

## References

[B1] Anders S. Huber W. (2010). Differential expression analysis for sequence count data. *Genome Biol.* 11:R106. 10.1186/gb-2010-11-10-r106 20979621 PMC3218662

[B2] Andrés F. Porri A. Torti S. Mateos J. Romera-Branchat M. García-Martínez J. L. (2014). SHORT VEGETATIVE PHASE reduces gibberellin biosynthesis at the *Arabidopsis* shoot apex to regulate the floral transition. *Proc. Natl. Acad. Sci. U.S.A.* 111 2760–2769. 10.1073/pnas.1409567111 24979809 PMC4084417

[B3] Angenent G. (1995). A novel class of MADS box genes is involved in ovule development in petunia. *Plant Cell* 7 1569–1582. 10.1105/tpc.7.10.1569 7580252 PMC161013

[B4] Arora R. Agarwal P. Ray S. Singh A. K. Singh V. P. Singh V. P. (2007). MADS-box gene family in rice: genome-wide identification, organization and expression profiling during reproductive development and stress. *BMC Genomics* 8:242. 10.1186/1471-2164-8-242 17640358 PMC1947970

[B5] Bowman J. L. Meyerowitz E. M. (1991). Genetic control of pattern formation during flower development in Arabidopsis. *Symp. Soc. Exp. Biol.* 45 89–115. 1688210

[B6] Chen Z. Rao P. Yang X. Su X. Zhao T. Gao K. (2018). A global view of transcriptome dynamics during male floral bud development in Populus tomentosa. *Sci. Rep.* 8:722. 10.1038/s41598-017-18084-5 29335419 PMC5768756

[B7] Cheng Z. Ge W. Li L. Hou D. Ma Y. Liu J. (2017). Analysis of MADS-Box gene family reveals conservation in floral organ ABCDE model of moso bamboo (*Phyllostachys edulis*). *Front. Plant Sci.* 8:656. 10.3389/fpls.2017.00656 28515730 PMC5413564

[B8] Coen E. S. Meyerowitz E. M. (1991). The war of the whorls: genetic interactions controlling flower development. *Nature* 353 31–37. 10.1038/353031a0 1715520

[B9] Ditta G. Pinyopich A. Robles P. Pelaz S. Yanofsky M. F. (2004). The SEP4 gene of Arabidopsis thaliana functions in floral organ and meristem identity. *Curr. Microbiol.* 14 1935–1940. 10.1016/j.cub.2004.10.028 15530395

[B10] Fan H. Chen L. (2006). Current distribution of endangered mangrove *Lumnitzera littorea* (Jack.) Voigt in China. *Guangxi Sci.* 13 226–227. 10.13656/j.cnki.gxkx.2006.03.019

[B11] Favaro R. Pinyopich A. Battaglia R. Kooiker M. Borghi L. Ditta G. (2003). MADS-box protein complexes control carpel and ovule development in *Arabidopsis*. *Plant Cell* 15 2603–2611. 10.1105/tpc.015123 14555696 PMC280564

[B12] Geuten K. Becker A. Kaufmann K. Caris P. Janssens S. Viaene T. (2006). Petaloidy and petal identity MADS-box genes in the balsaminoid genera Impatiens and Marcgravia. *Plant J.* 47 501–518. 10.1111/j.1365-313X.2006.02800.x 16856983

[B13] Grabherr M. G. Haas B. J. Yassour M. Levin J. Z. Thompson D. A. Amit I. (2011). Full-length transcriptome assembly from RNA-Seq data without a reference genome. *Nat. Biotechnol.* 29 644–652. 10.1038/nbt.1883 21572440 PMC3571712

[B14] Huang F. Xu G. Chi Y. Liu H. Xue Q. Zhao T. (2014). A soybean MADS-box protein modulates floral organ numbers, petal identity and sterility. *BMC Plant Biol.* 14:89. 10.1186/1471-2229-14-89 24693922 PMC4021551

[B15] Huang J. Z. Lin C. P. Cheng T. C. Chang B. C. Cheng S. Y. Chen Y.-W. (2015). A de novo floral transcriptome reveals clues into Phalaenopsis orchid flower development. *PLoS One* 10:e0123474. 10.1371/journal.pone.0123474 25970572 PMC4430480

[B16] Hui W.-K. Wang Y. Chen X.-Y. Zayed M. Z. Wu G.-J. (2018). Analysis of transcriptional responses of the inflorescence meristems in *Jatropha curcas* following gibberellin treatment. *Int. J. Mol. Sci.* 19:432. 10.3390/ijms19020432 29389867 PMC5855654

[B17] Irish V. F. (2010). The flowering of Arabidopsis flower development. *Plant J.* 61 1014–1028. 10.1111/j.1365-313X.2009.04065.x 20409275

[B18] Jin J. Zhang H. Kong L. Gao G. Luo J. (2014). PlantTFDB 3.0: a portal for the functional and evolutionary study of plant transcription factors. *Nucleic Acids Res.* 42 D1182–D1187. 10.1093/nar/gkt1016 24174544 PMC3965000

[B19] Kumar S. Stecher G. Li M. Knyaz C. Tamura K. (2018). MEGA X: molecular evolutionary genetics analysis across computing platforms. *Mol. Biol. Evol.* 35 1547–1549. 10.1093/molbev/msy096 29722887 PMC5967553

[B20] Li D. Liu C. Shen L. Wu Y. Chen H. Robertson M. (2008). A repressor complex governs the integration of flowering signals in *Arabidopsis*. *Dev. Cell* 15 110–120. 10.1016/j.devcel.2008.05.002 18606145

[B21] Li W. Zhang L. Ding Z. Wang G. Zhang Y. Gong H. (2017). De novo sequencing and comparative transcriptome analysis of the male and hermaphroditic flowers provide insights into the regulation of flower formation in and romonoecious Taihangia rupestris. *BMC Plant Biol.* 17:54. 10.1186/s12870-017-0990-x 28241786 PMC5329940

[B22] Li Y. H. Yang Y. Zhang Y. (2016). Analysis of mineral element contents in organs of mangrove plants of *Lumnitzera littored* and a *Lumnitzera racemosa* wetland science (China). *China Acad. J.* 14 433–438. 10.13248/j.cnki.wetlandsci.2016.03.020

[B23] Litt A. Kramer E. M. (2010). The ABC model and the diversification of floral organ identity. *Semin. Cell Dev. Biol.* 21 129–137. 10.1016/j.semcdb.2009.11.019 19948236

[B24] Liu B. Ou C. Chen S. Cao Q. Zhao Z. Miao Z. (2019). Differentially expressed genes between carrot petaloid cytoplasmic male sterile and maintainer during floral development. *Sci. Rep.* 9:17384. 10.1038/s41598-019-53717-x 31757985 PMC6874560

[B25] Livak K. J. Schmittgen T. D. (2001). Analysis of relative gene expression data using real-time quantitative PCR and the 2-△△CTmethod. *Methods* 25 402–408. 10.1006/meth.2001.1262 11846609

[B26] Luo M. Luo M.-Z. Buzas D. Finnegan J. Helliwell C. Dennis E. S. (2008). UBIQUITIN-SPECIFIC PROTEASE 26 is required for seed development and the repression of PHERES1 in Arabidopsis. *Genetics* 180 229–236. 10.1534/genetics.108.091736 18723879 PMC2535677

[B27] Mateos J. L. Madrigal P. Tsuda K. Rawat V. Richter R. Romera-Branchat M. (2015). Combinatorial activities of SHORT VEGETATIVE PHASE and FLOWERING LOCUS C define distinct modes of flowering regulation in *Arabidopsis*. *Genome Biol.* 16:31. 10.1186/s13059-015-0597-1 25853185 PMC4378019

[B28] Mertens A. Brys R. Schouppe D. Jacquemyn H. (2018). The impact of floral morphology on genetic differentiation in two closely related biennial plant species. *AoB Plants* 10:ly051. 10.1093/aobpla/ply051 30323915 PMC6178171

[B29] Mohanty J. N. Joshi R. K. (2018). Molecular cloning, characterization and expression analysis of MADS-box genes associated with reproductive development in Momordica dioica Roxb. *3 Biotech* 8:150. 10.1007/s13205-018-1176-4 29616182 PMC5866819

[B30] Moon J. Suh S. S. Lee H. Choi K. R. Hong C. B. Paek N.-C. (2003). The SOC1 MADS-box gene integrates vernalization and gibberellin signals for flowering in *Arabidopsis*. *Plant J.* 35 613–623. 10.1046/j.1365-313x.2003.01833.x 12940954

[B31] Parenicova L. De Folter S. Kieffer M. Horner D. S. Favalli C. Busscher J. (2003). Molecular and phylogenetic analyses of the complete MADS-box transcription factor family in *Arabidopsis*: new openings to the MADS world. *Plant Cell* 15 1538–1551. 10.1105/tpc.011544 12837945 PMC165399

[B32] Polidoro B. A. Carpenter K. E. Collins L. Duke N. C. Ellison A. M. Ellison J. C. (2010). The loss of species: mangrove extinction risk and geographic areas of global concern. *PLoS One* 5:e10095. 10.1371/journal.pone.0010095 20386710 PMC2851656

[B33] Rocheta M. Sobral R. Magalhaes J. Amorim M. I. Ribeiro T. Pinheiro M. (2014). Comparative transcriptomic analysis of male and female flowers of monoecious Quercus suber. *Front. Plant Sci.* 5:599. 10.3389/fpls.2014.00599 25414713 PMC4222140

[B34] Rosas-Guerrero V. Hernandez D. Cuevas E. (2017). Influence of pollen limitation and inbreeding depression in the maintenance of incomplete dichogamy in Salvia elegans. *Ecol. Evol.* 7 4129–4134. 10.1002/ece3.2827 28649325 PMC5478062

[B35] Scortecci K. C. Michaels S. D. Amasino R. M. (2001). Identification of a MADS-box gene, FLOWERING LOCUS M, that represses flowering. *Plant J.* 26 229–236. 10.1046/j.1365-313x.2001.01024.x 11389763

[B36] Streisfeld M. A. Young W. N. Sobel J. M. (2013). Divergent selection drives genetic differentiation in an R2R3-MYB transcription factor that contributes to incipient speciation in Mimulus aurantiacus. *PLoS Genet.* 9:e1003385. 10.1371/journal.pgen.1003385 23555295 PMC3605050

[B37] Su G. H. (2004). *Study on Genetic Diversity in Lumnitzera of Mangrove.* Ph. D. Thesis, Sun Yat-sen University, Guangzhou.

[B38] Theissen G. Melzer R. Ruempler F. (2016). MADS-domain transcription factors and the floral quartet model of flower development: linking plant development and evolution. *Development* 143 3259–3271. 10.1242/dev.134080 27624831

[B39] Wang Y. Li L. Ye T. Lu Y. Chen X. Wu Y. (2013). The inhibitory effect of ABA on floral transition is mediated by ABI5 in *Arabidopsis*. *J. Exp. Bot.* 64 675–684. 10.1093/jxb/ers361 23307919 PMC3542054

[B40] Wellmer F. Graciet E. Luis Riechmann J. (2014). Specification of floral organs in *Arabidopsis*. *J. Exp. Bot.* 65 1–9. 10.1093/jxb/ert385 24277279

[B41] Yun H. Hyun Y. Kang M. J. Noh Y. S. Noh B. Choi Y. (2011). Identification of regulators required for the reactivation of FLOWERING LOCUS C during *Arabidopsis* reproduction. *Planta* 234 1237–1250. 10.1007/s00425-011-1484-y 21773790

[B42] Zhang Q. Liu L. Zhu F. Ning Z. Hincke M. T. Yang N. (2014). Integrating de novo transcriptome assembly and cloning to obtain chicken Ovocleidin-17 full-length cDNA. *PLoS One* 9:e93452. 10.1371/journal.pone.0093452 24676480 PMC3968166

[B43] Zhang X. N. Zhong C. R. Yan T. L. Zhang Y. (2016). The germplasm resource rescue of endangered mangrove (Lumnitzera littorea (Jack.) Voigt) by artificial pollination. *Ecol. Sci.* 35 38–42. 10.14108/j.cnki.1008-8873.2016.05.006

[B44] Zhang Y. Li Y. H. Zhang X. N. Yang Y. (2017). Flower phenology and breeding system of endangered mangrove Lumnitzera littorea (Jack.) Voigt. *Chin. J. Appl. Environ. Biol.* 23 77–81. 10.3724/SP.J.1145.2016.03021

[B45] Zhang Y. Zhong C. Li S. Yan T. Guan W. (2013). Endangered species of mangrove plants: *Lumnitzera littore*. *Forest Resour. Manag.* 5 103–107. 10.13466/j.cnki.lyzygl.2013.05.026

[B46] Zhang Y. Zhong C. R. Yang Y. Zhong H. B. Zeng Z. P. Zhang J. (2018). Rescue of germplasm resources of endangered mangrove plant *Lumnitzera littorea*. *Mol. Plant Breed.* 14 4112–4118. 10.13271/j.mpb.016.004112

[B47] Zhong C. R. Li S. C. Guan W. Li H. L. Lin X. Y. Bao-wen L. (2011). Current distributions of three endangered mangrove species in China. *Ecol. Sci.* 30 431–435.

[B48] Zhou Q. J. Chen Y. M. Wu W. Zhou R. Z. Zhang Y. (2018). The complete chloroplast genome sequence of an endangered mangrove tree *Lumnitzera littorea* (Combretaceae). *Conserv. Genet. Resour.* 10 911–913. 10.1007/s12686-017-0929-4

