# Supplementary figures and images for: Comparative Transcriptome Reveals the Genes’ Adaption to Herkogamy of Lumnitzera littorea (Jack) Voigt
Source: Front Genet. 2020 Dec 8;11:584817. doi: 10.3389/fgene.2020.584817 (PMC7753066; doi:10.3389/fgene.2020.584817)

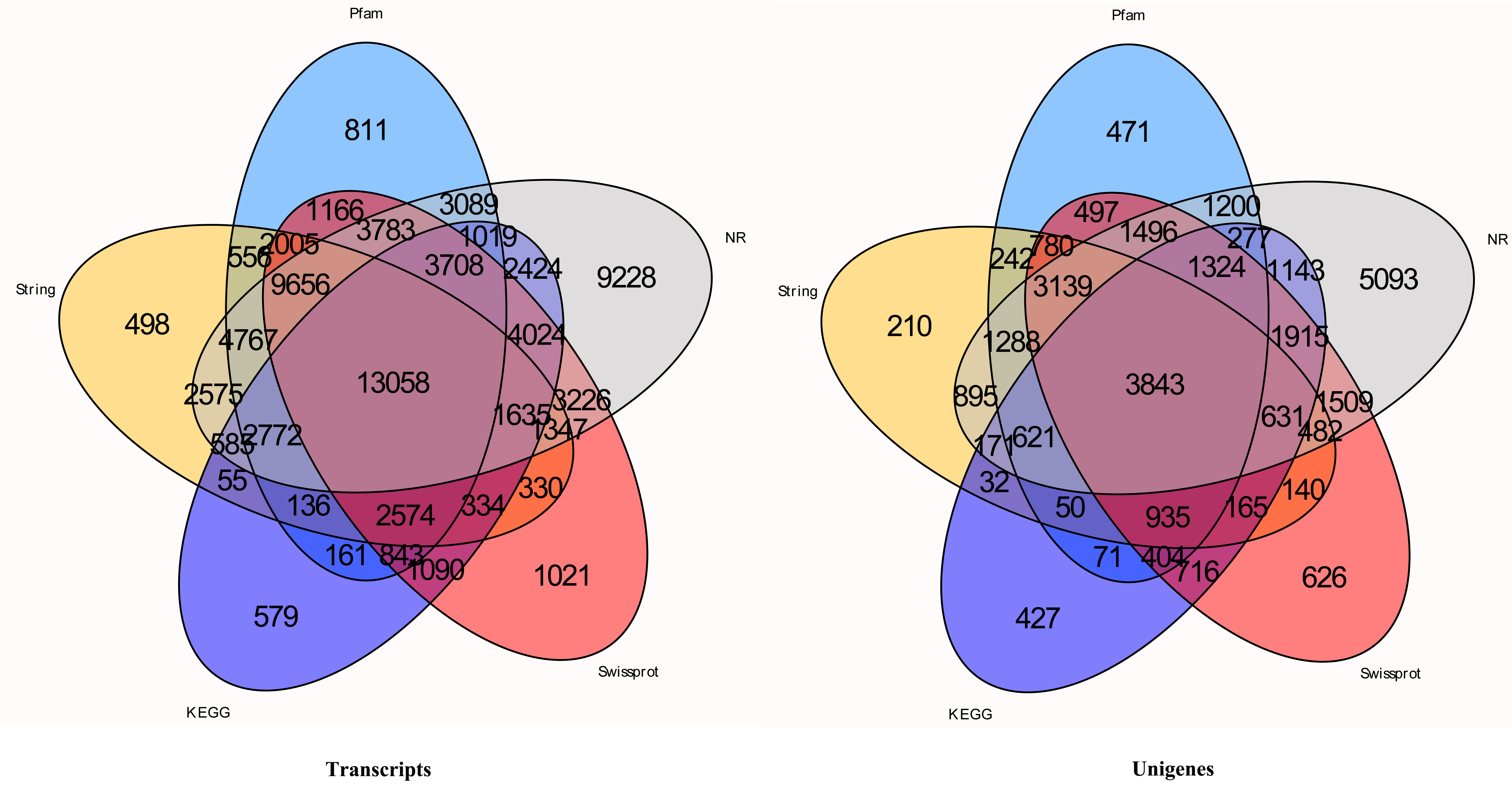

Supplement: Supplementary Figure 1 — Transcript and unigene annotation with the NCBI NR, SwissProt, String, KEGG and Pfam databases of L. littorea transcriptome data. [file Image_1.JPEG]

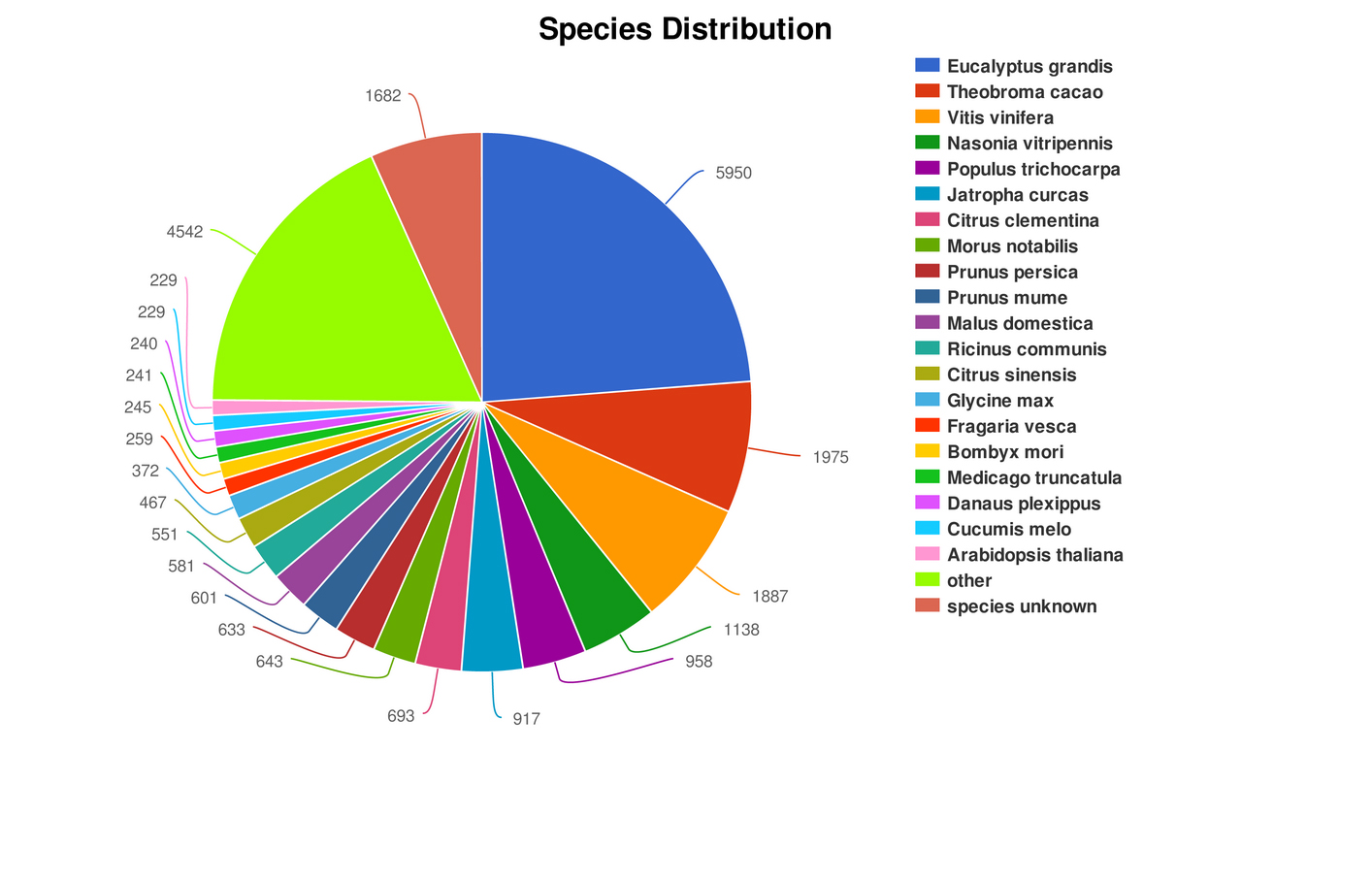

Supplement: Supplementary Figure 2 — Species distribution with BLAST hits to the annotated unigenes of L. littorea. [file Image_2.JPEG]

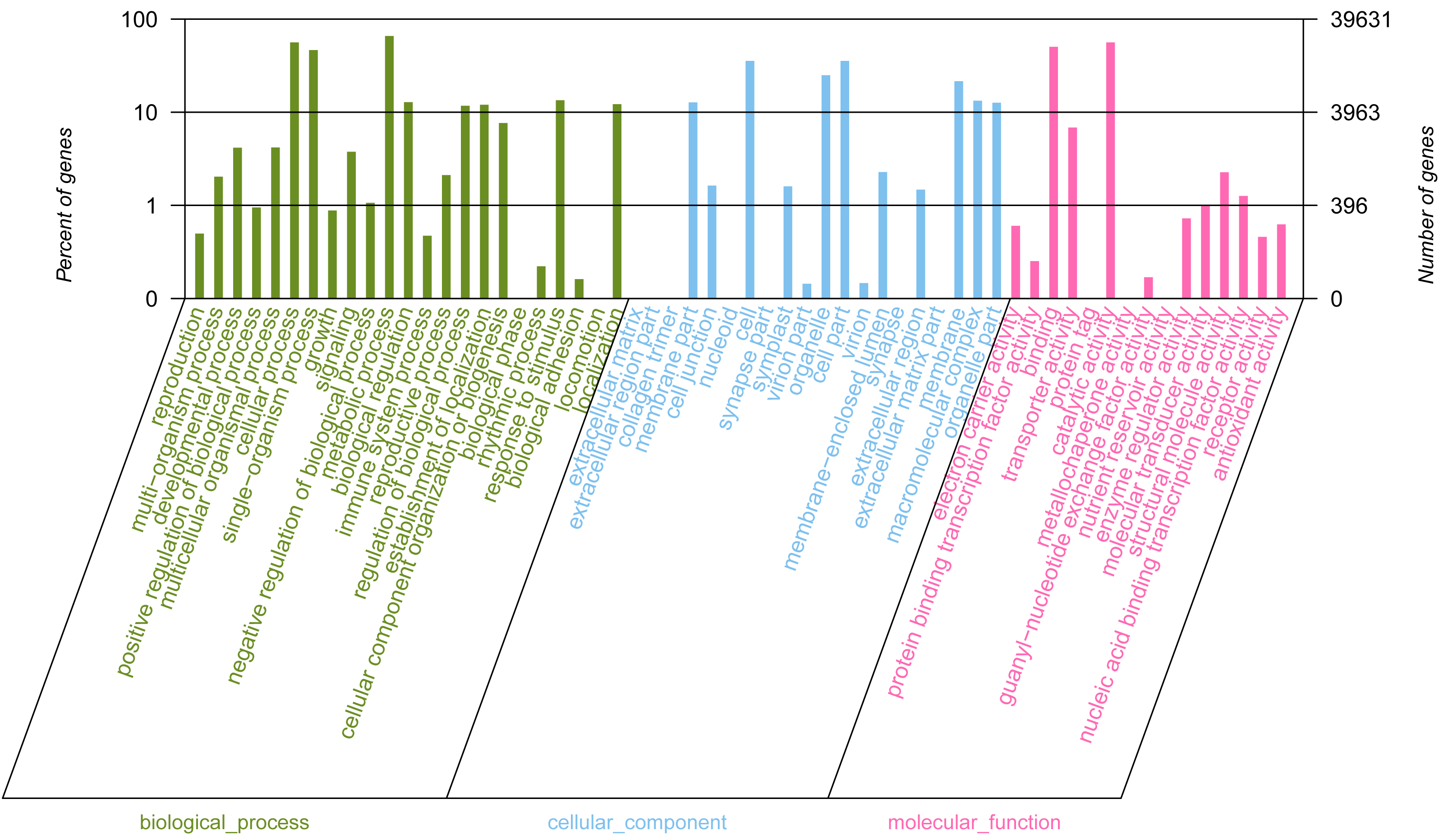

Supplement: Supplementary Figure 3 — GO classification summarized by three main categories: Biological process, cellular component, and molecular function. [file Image_3.JPEG]

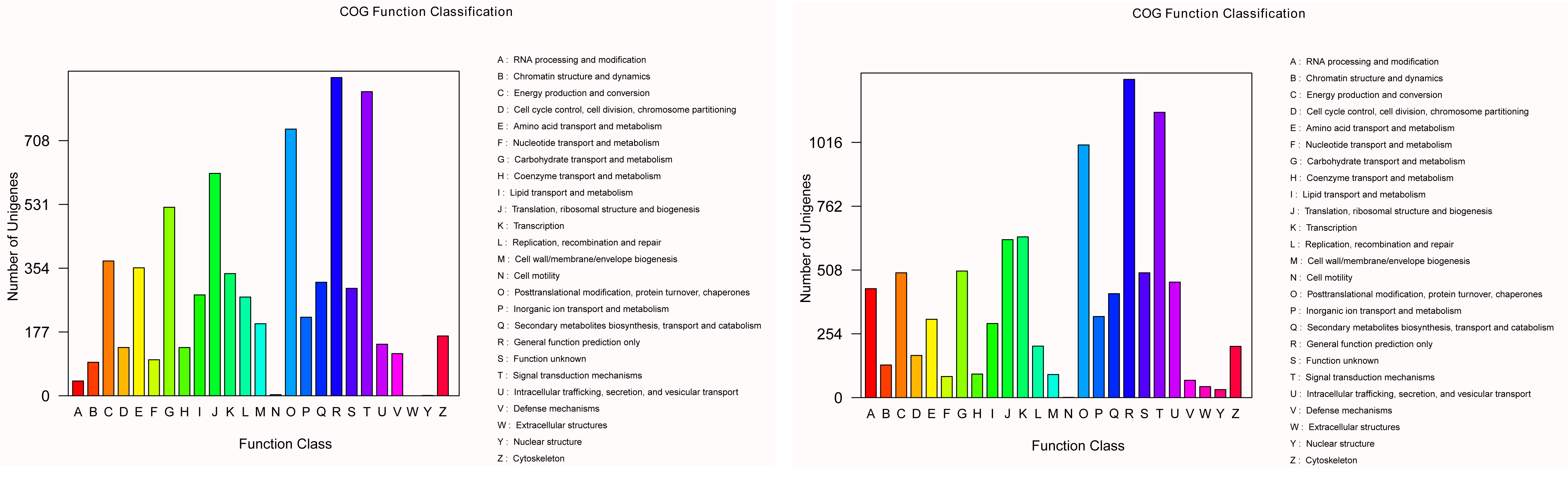

Supplement: Supplementary Figure 4 — COG and KOG functional classification of the unigenes of L. littorea with NR annotation. [file Image_4.JPEG]

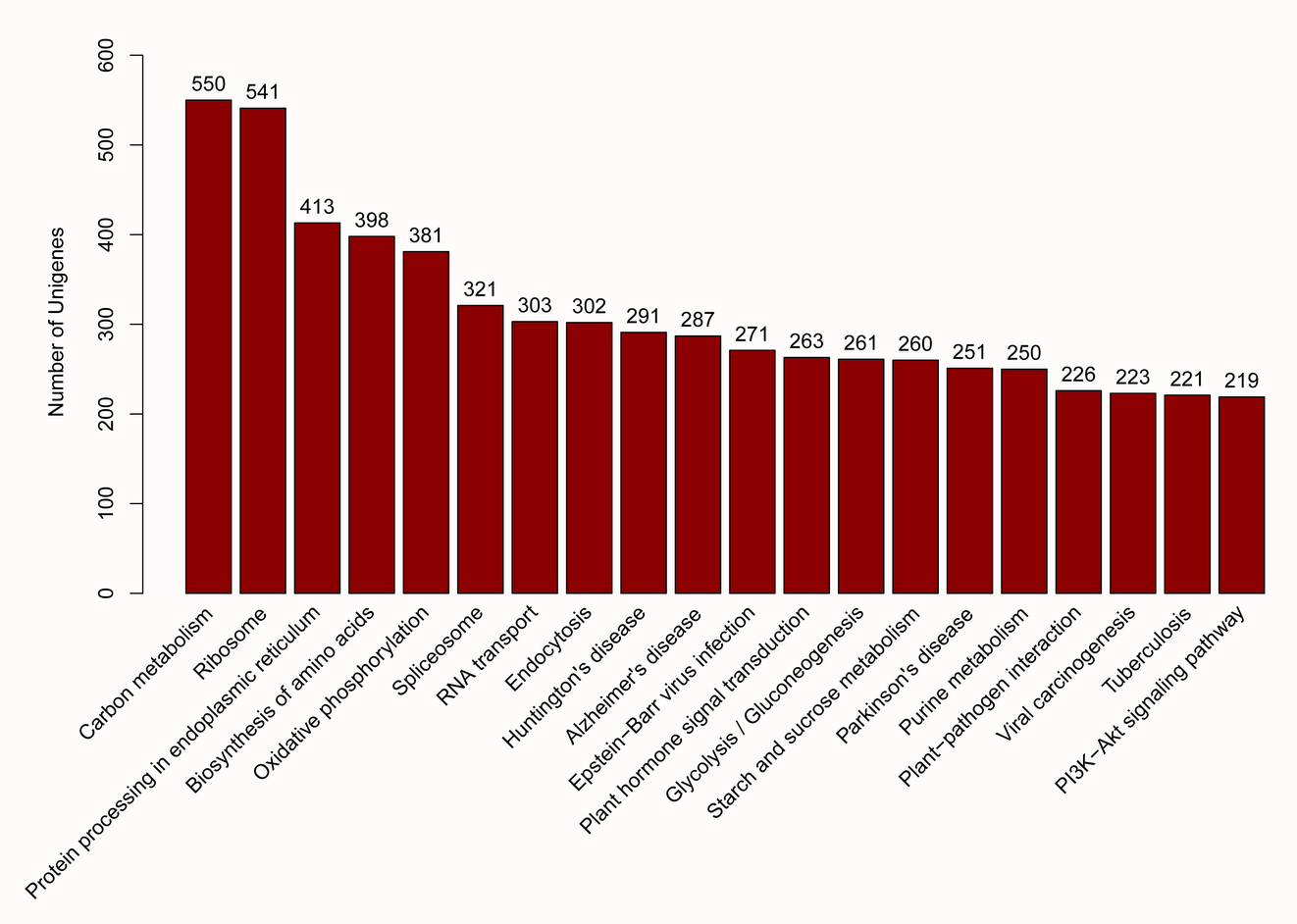

Supplement: Supplementary Figure 5 — The biological pathways of L. littorea flower unigenes against the KEGG database. [file Image_5.JPEG]

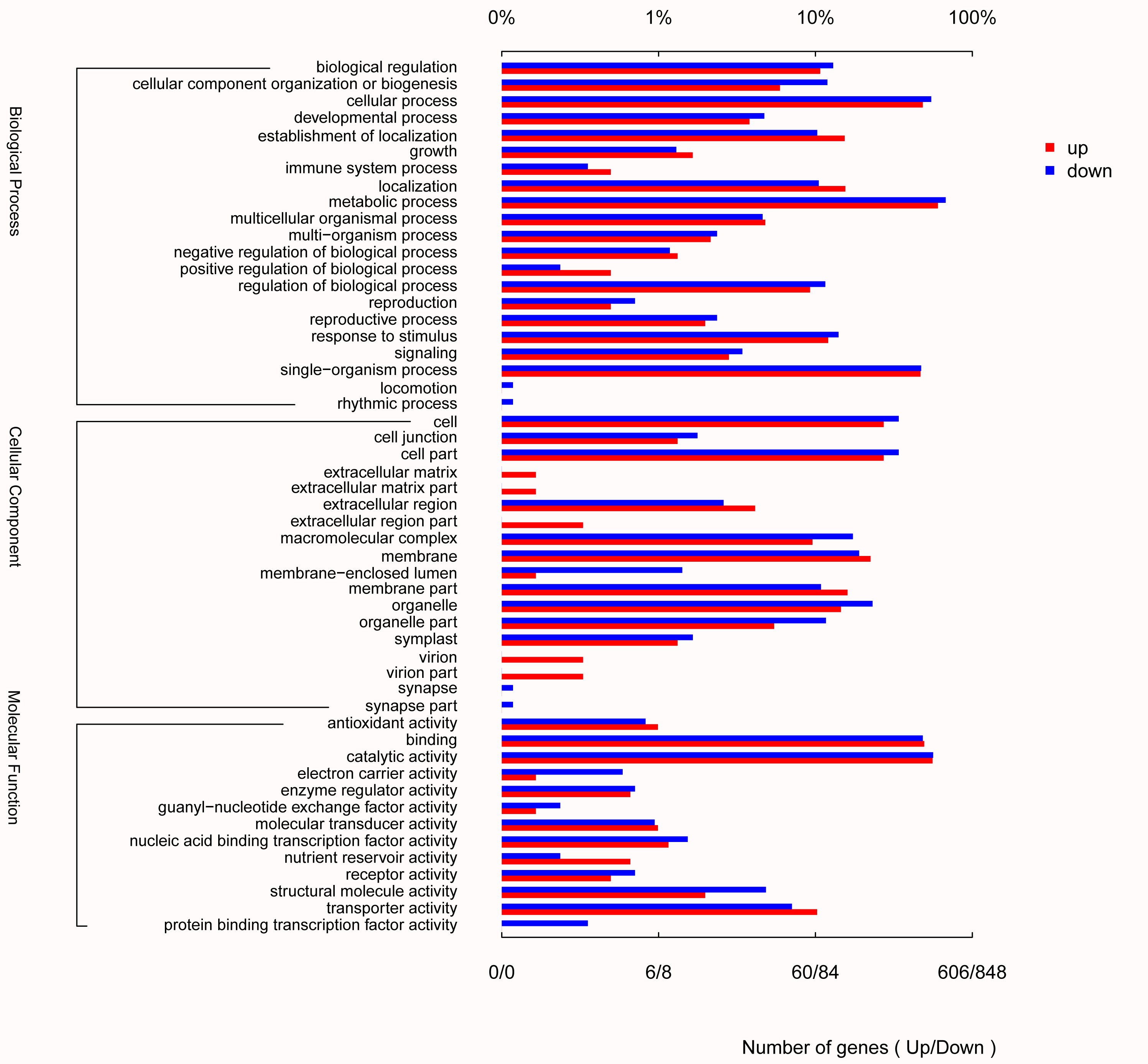

Supplement: Supplementary Figure 6 — DEGs in different column development stages between L-1 and L-2. [file Image_6.JPEG]

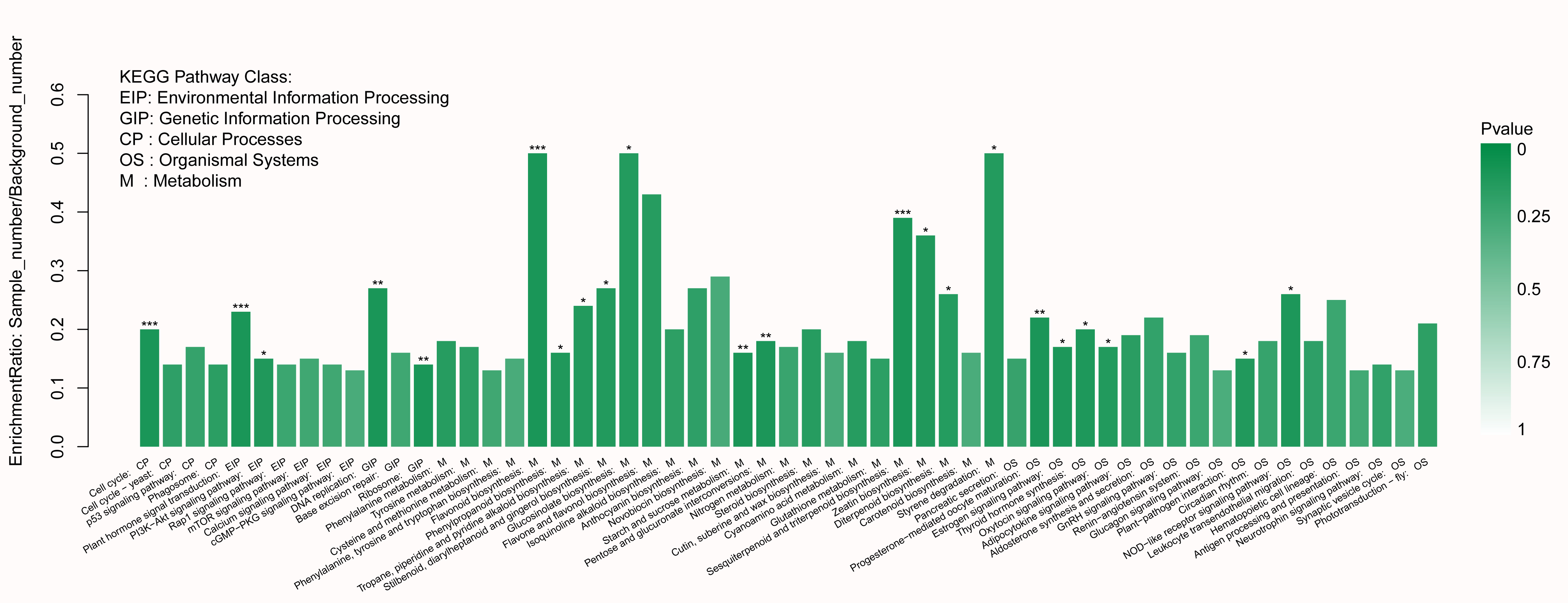

Supplement: Supplementary Figure 7 — Functional KEGG pathway annotation of the DEGs of L. littorea. [file Image_7.JPEG]
